# Notes and News

**Published:** 1888-06-16

**Authors:** 


### Notes and News.

Collected and Communicated.

A new home for consumptives has recently been opened in Brooklyn. It contains forty-two wards, in each of which there are from one to eight beds. The cost of the building is estimated at £14,000.

John Sillick, a young man who was to have been married in a few days, committed suicide by leaping from the Clifton Suspension Bridge, a depth of 240 feet. This is the twenty-sixth suicide in twenty-four years at the same place.

The nurses of the Torbay Hospital were last week, by the kindness of Mrs. Mallock, Cockington Court (wife of Mr. Mallock, M.P.), entertained at afternoon tea. They have also been able, through the generosity of Miss Desborough, to enjoy a day on the River Dart.

A special meeting of The Hospitals Association will be held at the Governors' Hall, St. Thomas's Hospital, S.E., on Wednesday, June 20th, at eight p.m., for the adjourned discussion of a paper by Mr. W. Burdett-Coutts, M.P., on "Contributions by Patients in Relation to the Financial Condition of London Hospitals." The chair will be taken by the President, Dr. J. S. Bristowe. Cards of admission can be obtained on application from Mr. L. Broke Willoughby, Secretary, The Hospitals Association, Norfolk House, Norfolk Street, W.C.

The London Samaritan Society (which has its headquarters at High Street, Homerton) has just established its third convalescent home in Sandgate, and the new premises, known as "The Homestead," were formally opened on Saturday evening by General Sir Charles and Lady Keyes. The new home is a convenient and commodious building, it stands in pleasant grounds, overlooks a wide stretch of ocean, and is close to the sea; while it is sheltered from the northerly as well as the easterly winds. Altogether it is most admirably situated for the purposes to which it is intended to be put. The dedicatory service was the occasion of an interesting gathering on the lawn of the Home.

The annual meeting of subscribers to the Manchester and Salford Lock and Skin Diseases Hospital was held on Wednesday, at the hospital, Duke-street, Liverpool Road, Mr. Councillor George Clay presiding. Mr. R. C. Stonex, the secretary, presented a financial statement, which showed that the balance in the bank in 1886 (the accounts being for two years) was £106. The annual subscriptions for 1887 amounted to £288, donations and legacies to £1,200, proportion of Hospital Sunday and Saturday Fund £138, and sundry receipts £167, making a total of £1,797. After meeting all necessary expenses there remained a balance in the bank of £684. During the last two years the number of patients treated were:—Males, 4,390; females, 1,415; and children 285, making a total of 6,090. Out of 306 in-patients who had left the wards since the last report, only twenty-two could be traced as not having been restored to their friends or sent to charitable homes. The committee had decided to recommend that the skin diseases department, which is now situated in Dale-street, Piccadilly, should henceforth be conducted as a separate institution.

The weather has not yet been found excessively hot by the average mortal, but it has proved too much for the British dock labourer. There are now many vessels lying in London docks waiting for labourers who will condescend to relieve them of their cargo, and waiting in vain. Six or seven months hence the scene will be changed. Life is a more costly business in winter than in summer, and in December the very men who are now idle by their own free will, will be posing as the starving unemployed of Trafalgar Square, the casual wards, and the charity relief funds. It is facts like these that tire the patience and faith of the man who wants to help his poorer neighbours, and give an excuse to him to say, "Heaven helps those that help themselves," and to leave Heaven to see to it unaided.

A Justice of the Peace, who is one of the most influential governors of a representative provincial hospital, expresses a sound opinion in a letter we have just received. He declares Lord Randolph Churchill was quite right when he said that annual subscriptions were the backbone of hospital revenues in this country, and if London folk have not the wisdom to subscribe to the metropolitan hospitals in the same proportion that country people do to their local institutions, they will sooner or later have to pay a hospital rate and to put up with all the waste, jobbery, and numerous other evils which such an expenditure of public money will surely entail. These are weighty words, sound, accurate, and true. We commend them, with the experience of that rate-supported institution, the Eastern Hospital, of disreputable memory, to the careful consideration of all classes of the metropolitan public.

In and around Gravesend the inhabitants will in future not only be able to obtain the services of trained nurses at their own homes by application to the Gravesend Hospital, as heretofore, but the Committee have decided to "provide district nurses for the nursing of the sick poor, and for the purpose of carrying on this work of mercy they will employ nurses, and make such rules and regulations with reference thereto as they may deem fit." Such a resolution on the part of a hospital committee implies both a clear comprehension of the urgent necessities of the poor in times of sickness, and an intelligent sense of duty. In a small, poor, and unsanitary dwelling medical attendance alone, in the event of serious illness, is little better than a mockery and a delusion. Of what avail is it for the doctor to see his patient and send him medicine if there be no one to carry out his instructions as to care, quiet, cleanliness, and good order, which are often a thousand times more important than any medicine can possibly be? We are gradually learning in this country that "drugging" is frequently but a very small part of skilled medical regimen. In the annual report just received it is announced that Mrs. John Russell has presented to the hospital, as a jubilee offering, two new wards. The new wards have enabled the committee to carry out their enlightened and benevolent scheme of district nursing. By means of a bazaar and the efforts of the ladies of the town and district, the "Russell Wards" have been fully furnished, and are now ready for use. In spite of all this good work on the part of the management, and of Mrs. John Russell's liberality, we regret to see a deficit in the year's accounts of nearly £50. The public should feel bound to support the managers more zealously.

THE following vacancies are announced this week, the amount of salary in each case being given after the name of the institution :—

**Resident Medical Officers.**

East London Hospital for Children, Shadwell, E. Resident Clinical Assistant.—Board ; no salary.
Guest Hospital, Dudley. Resident Medical Officer.—£110 per annum, with board, etc.
Metropolitan Asylums Board Western Fever Hospital, Fulham, S.W. Clinical Assistant.—Board ; no salary.
North Eastern Hospital for Children, Hackney Road, E. Junior House Surgeon.—£30 for six months.
Parish of Lochs, Stornoway. Medical Officer of Health.—£140, with house and rates free
Ramsgate and St. Lawrence Royal Dispensary and Seamen's Infirmary. Resident Medical Officer.—£120 per annum, and £10 for substitute during annual holiday.
Royal Albert Hospital, Devonport. Assistant House Surgeon.—Board ; no salary.
Royal South Hants Infirmary, Southampton. House Surgeon.—£100, and board.
Sheffield General Infirmary. House Surgeon.—£120 per annum, with board.
Sheffield General Infirmary. Assistant House Surgeon.—£80 per annum, with board.
Surrey Dispensary, Great Dover Street, Southwark. House Surgeon.—£120 per annum.
Wellingborough and District Medical Institute. Medical Officer. £280, and fees, with dwelling house.
West London Hospital, Hammersmith Road, W. House Physician. Board ; no salary.
West London Hospital, Hammersmith Road, W. House Surgeon. Board ; no salary.
Wolverhampton and Staffordshire General Hospital. Resident Assistant.—Board ; no salary.

**Non-resident Medical Officers.**

Anderson's College Dispensary, Glasgow. Physician.
Anderson's College Dispensary, Glasgow. Surgeon.
Board of Works for the Wandsworth District. Medical Officer for the parish of Clapham.—£75 per annum, with increase.
Celbridge Union. Medical Officer for the Workhouse.—£100 per annum, and £15 per annum as Medical Superintendent Officer of Health.
Duffus Parochial Board. Medical Officer.—£35 per annum.
Evelina Hospital for Sick Children, Southwark Bridge Road. Surgeon for Out patients.
King's College Hospital. Assistant Surgeon.
London Temperance Hospital, Hampstead Road, N.W. Surgeon.
Newton Heath (Manchester) District. Medical Officer of Health. —£50 per annum.
Parishes of Pennygorm and Torosay. Medical Officer.—£100 per annum.
West London Hospital, Hammersmith Road, W. Physician.
West London Hospital, Hammersmith Road, W. Surgeon.
Westport Union. Medical Officer, Westport No. 2 and Louisburgh No. 2 districts.—£39 per annum, and fees.

---

A CASUAL Ward for Women and Children was opened at the Rue Latat, Paris, by M. Jules Simon on the 2nd inst. It is called the Hartmann Ward, having been founded by the millionaire, M. Hartmann, and twenty-four charitable friends. It is large enough to receive eight hundred inmates, and there are seventy beds in each dormitory. One dormitory is intended for women who have babies, and is provided with a cradle at the side of each bed. The bath-rooms, kitchens, etc., are admirable. Though one cannot but feel a sense of sadness in recalling the fact that such refuges are needed, it is satisfactory to note the care shown by the richer inhabitants of Paris for their homeless sisters.

---

WE observe in the report for 1887 of the Longmore Hospital for Incurables, Edinburgh, which has just been published, that the committee are earnestly appealing for increased subscriptions. It is much to be regretted that so valuable an institution should be allowed to be in need ; but it would appear that for the last two years the funds have been decreasing, while the expenses have been increasing—with the result that for last year a loss of something like £1,000 has been suffered. We would express the hope that the committee of so practical a charity as the Longmore Hospital will be able in future to carry on their benevolent labours unhampered by any lack of funds.

THE Right Rev. Dr. Kingdon, Bishop (Coadjutor) of Fredericton, will preach on Sunday, June 17th, on behalf of the Hospital Fund, at eleven o'clock, in Canon Ingram's church, St. Margaret's, Lothbury, near the Bank of England.

---

THE Rev. Dr. Forrest, of St. Jude's, S.W., has by sheer hard work supplanted Canon Fleming in the proud position he has so long occupied as the largest contributor to the Hospital Sunday Fund, by raising this year the splendid sum of £1,159 on Hospital Sunday.

---

THE Belgian burgomasters and aldermen who have been in the metropolis on a visit to the Lord Mayor, on leaving London on their return to Belgium, besides the £100 they have subscribed to the British Home for Incurables, handed to the Lord Mayor a cheque for £50 for the Belgian Benevolent Society in London.

---

THE annual meeting of the After-Care Association for Poor and Friendless Female Convalescents on Leaving Asylums for the Insane will take place at 83, Lancaster Gate, W., on Wednesday, July 4th, at three p.m. The Earl of Meath will preside. Cards of invitation can be obtained of the Secretary, Mr. H. Thornhill Roxby, Emblewood, Osbaldeston Road, London, N.

---

THE *Times* states that the success of the National Pension Fund for Nurses is now assured. At a meeting of the council on Thursday upwards of 120 policies were accepted and 326 proposals were reported. One thousand six hundred nurses have already applied at the office for forms of proposal to fill up, and similar applications are being made in increasing numbers every week. The authorities of several of the chief hospitals and nursing institutions are manifesting a warm interest in the scheme, and to meet them as far as possible the council have determined to establish trust funds in the name of such institutions as become affiliated with the fund by agreeing to pay the whole or a portion of the premiums of the nurses whom they employ, and who remain in their service for a period of years. The council have also agreed to allow 3 per cent. interest upon all the money paid into the fund pending the issue of the first 1,000 policies. Lady Rosebery has given £25 to the bonus fund.

---

THE political economists declare that it is contrary to the laws of Nature and the science to which they have devoted their energies for women to go out to work. Women's work lowers men's wages, they say, and thence argue (being for the most part of the male sex) that women should refrain from money-making. Unfortunately, their gospel is traversed by the law of physiology which says that neither man nor woman can live without food, and the law of circumstance which places so many women in positions where they must work or starve. This bears especially cruelly on those women, married or widows, who have children dependent on them, whose bread they must go out to earn. The number of accidents that happen every year to young children left to the care of chance while their mothers are out working is almost countless, and to both mothers and children a crèche, or day nursery, where the babies will be looked after and kept out of mischief is an immense boon. The Southsea Infant Nursery, from which we have just received a report, has now been in existence ten years, during which time there have been 68,685 attendances. The daily average of attendances last year was twenty-nine—quite enough for the personal care that makes such an institution really effective—though on one occasion the admissions numbered forty-one. Besides these day-boarders there are nineteen resident children, some orphans, some whose mothers are in service, and one whose parents have deserted her. The ladies of the committee, of which Lady Willis is president, take a personal interest in the children at the crèche, who, as a rule, improve in health when they are regular attendants, and receive the advantages of regular care, instruction, amusement, and food.

